# The outcomes of Perthes’ disease of the hip: a study protocol for the development of a core outcome set

**DOI:** 10.1186/s13063-018-2695-3

**Published:** 2018-07-13

**Authors:** Donato Giuseppe Leo, Wei Yee Leong, Tina Gambling, Andrew Long, Rebecca Murphy, Helen Jones, Daniel Christopher Perry

**Affiliations:** 1 School of Sport & Exercise Sciences, Liverpool John Moores University, Liverpool, UK; 2 Alder Hey Children’s Hospital, Prescott Road, Liverpool, L14 2AB UK; 3 School of Healthcare Sciences, Cardiff University, Cardiff, UK; 4 School of Healthcare, University of Leeds, Leeds, UK; 5 Institute of Translational Medicine, Institute in the Park, University of Liverpool, Prescott Road, Liverpool, L14 2AB UK

**Keywords:** Core outcomes set, Delphi, Consensus outcomes, Perthes’ disease, Legg-calve-Perthes’ disease

## Abstract

**Background:**

Perthes’ disease is an idiopathic osteonecrosis of a developmental hip that is most frequent in Northern Europe. Currently, the absence of a common set of standardised outcomes makes comparisons between studies of different interventions challenging. This study aims to summarise the outcomes used in clinical research of interventions for Perthes’ disease and define a set of core outcomes (COS) to ensure that the variables of primary importance are measured and reported in future research studies investigating Perthes’ disease.

**Methods:**

A systematic review of the current literature will be used to identify a list of outcomes reported in previous studies. Additional important outcomes will be sought by interviewing a group of children with Perthes’ disease, adults who were treated with the disease in infancy and parents of children with the disease. This list will then be evaluated by experts in Perthes’ disease using a Delphi survey divided into two rounds to ascertain the importance of each outcome. The final outcomes list obtained from the Delphi survey will be then discussed during a consensus meeting of representative key stakeholders in order to define the COS to be reported in future clinical trials related to Perthes’ disease.

**Discussion:**

The absence of high-quality research and clear guidelines concerning the management of Perthes’ disease is, at least in part, due to the difficulties in the comparing the results from previous studies. The development of a COS seeks to standardise outcomes collected in future research studies to enable comparisons between studies to be made and to facilitate meta-analyses of results.

**Trial registration:**

Core Outcome Measures in Effectiveness Trials Initiative (COMET), 1003. Registered on 20 July 2017. Prospero International Prospective Register of Systematic Reviews, CRD 42017069742. Registered on 10 July 2017.

**Electronic supplementary material:**

The online version of this article (10.1186/s13063-018-2695-3) contains supplementary material, which is available to authorized users.

## Background

Perthes’ disease is an idiopathic osteonecrosis of the hip in childhood. It most commonly affects boys aged 4–8 years [1]. The highest incidence of Perthes’ disease is in Northern Europe, particularly the Northern part of the UK [2] and Norway [3]. Perthes’ disease generates a susceptibility of the femoral head, to change shape, due to the forces acting across the joint [4, 5]. These shape changes alter the way that the joint moves, which can cause lifelong pain, functional limitations and accelerate the development of osteoarthritis [6].

Clinical treatments focus on the prevention of femoral head collapse, restoring the range of motion (ROM) and improving the functional recovery (absence of pain, amount of usual daily activity and sport-related activity) of the children [7]. Even though there are many published studies investigating the effectiveness of various surgical or non-surgical treatments, there is no consensus for the best management approach in the paediatric orthopaedic community [8]. In fact, there are no standard outcome methods to assess the success of treatment, which results in difficulties when trying to make comparisons between studies.

The absence of standard outcomes is one of the important pieces of feasibility information required before definitive intervention studies can begin. The development of core outcome sets (COS), popularised through the COMET Initiative, is the approach that has been developed to formulate a set of standardised outcomes particularly for use in clinical research such as randomised controlled trials (RCT) [9]. COS in clinical trials seek to reduce heterogeneity of the outcomes, reduce bias, improve the accuracy of data interpretation and allow meaningful comparisons between studies facilitating meta-analysis [9].

Currently, a small number of COS have been developed within orthopaedic surgery, such as for hip fractures [10] or on generic total joint replacement [11]. To date, no COS are available to determine the success of interventions used in the treatment of Perthes’ disease of the hip in childhood.

## Aim and objectives

### Aim

The aim of this study is to develop a COS for Perthes’ disease treatment in children, which can be used in clinical and cost effectiveness studies [9].

### Objectives

1. Systematically review the current literature to identify outcomes used in previous studies of interventions for Perthes’ disease;
2. Identify outcomes important to children and parents through an interview process;
3. Prioritise the outcomes from key stakeholders, such as surgeons, physiotherapists and family doctors using a Delphi survey;
4. Conduct a consensus meeting where the outcome list will be discussed with all stakeholders and parent and child representatives to form the core outcomes list.

## Methods/design

### Systematic review

The aims of the systematic review are to identify the primary and secondary outcomes in both operative and non-operative intervention strategies for Perthes’ disease. All RCTs, cohort studies and case series that include patients treated for Perthes’ disease, irrespective of their treatment type, that report childhood outcomes of the disease, will be included. Following the PICO (Population Intervention Comparison Outcomes) approach, the inclusion criteria are here summarised:

- Population: Children with Perthes’ disease
- Intervention and comparator: any treatment
- Outcomes: any outcomes

All studies must involve humans and all studies must be in the English language. This review will be limited to manuscripts in English, which have been published since 1990. The systematic review aims to generate a list of all outcomes measures used in the current literature.

#### Selection of studies

The search strategy will identify all published papers on the management of Perthes’ disease. Databases involved in the search will be the Cochrane Library, PubMed and Web of Science. Multiple databases will be used to maximise the sensitivity of the search strategy. The time period searched will be between January 1990 to January 2017.

#### Eligibility of studies

Studies will be selected by two reviewers (DGL and WJL) who will screen all the titles and abstracts. Titles of articles will be reviewed and included or excluded using Rayyan software [12]. Full text of all the manuscripts that match the inclusion criteria or manuscripts in which the abstract does not give enough information to make a clear decision about their inclusion will be obtained. This process will be documented with the PRISMA [13] flow diagram.

#### Data extraction

Data from eligible studies will be extracted through the data extraction form (Additional file 1: Appendix S1). This involves identification of the primary objective, prospective/retrospective data collection, study type, population, number of patients, conservative management, surgical management, primary and secondary outcomes measured, outcome assessment tools, follow-up.

#### Data analysis and presentation

All outcomes reported in eligible studies will be extracted and tabulated with their definition and measurement method(s) and then categorised in domains. To ensure the comprehensiveness of COS, outcomes terms will be assigned to one of the five core domains of the OMERACT [14] framework, that include the areas that should be covered by outcomes measures in order to ensure an adequate reporting of the results. The five domains of the OMERACT filter 2.0. are divided as: (1) adverse event; (2) life impact; (3) resource use; (4) pathophysiological manifestations; and (5) death. As suggested by Dorman et al. [15], the additional sixth domain of ‘technical considerations’, not included in the original OMERACT filter, will be included in order to assess technical or surgical outcomes that surgeons use to quantify successful outcomes. Under this domain will also be assessed the feasibility of use in clinical practice of the reported outcomes [16]. All six areas, related to the purpose of the review, are listed in Table 1.

**Table 1:** Modified OMERACT filter 2.0. core areas

Core area	Core domains	Example(s)
Adverse events	Adverse events	Unintended consequences
Life impact	Physical/Social/Emotional/ Cognitive/Health-Related quality of life	Quality of life, pain, impact on family, absence from school, participation in sports activities, functional scores – hip ROM and gait impairments
Resource use	Economic/Hospital/Need for intervention/Social burden	Length of stay, further surgery, physiotherapy
Pathophysiological manifestations	Musculoskeletal	Femoral head collapse, healing process, impingement
Death	N/A	N/A
Technical considerations	Technical/Surgical considerations	Radiographic measurement Feasibility of use in clinical practice

### Identification of key outcomes to patients and parents

#### Overview

Patients and parents’ opinions will be investigated and integrated in the COS development process through the identification of patient-reported outcomes (PROs) through semi-structured interviews administered to the patients and their parents, in order to assess the life impact of the disease [17]. Patient involvement is a fundamental step in defining the COS, following the COMET guidelines [9]. PROs identified through semi-structured interviews will be added to outcome list obtained from the systematic review. The full list will then be submitted for the experts’ evaluation through round 1 of the Delphi Survey.

#### Interview process

**Fig. 1:**
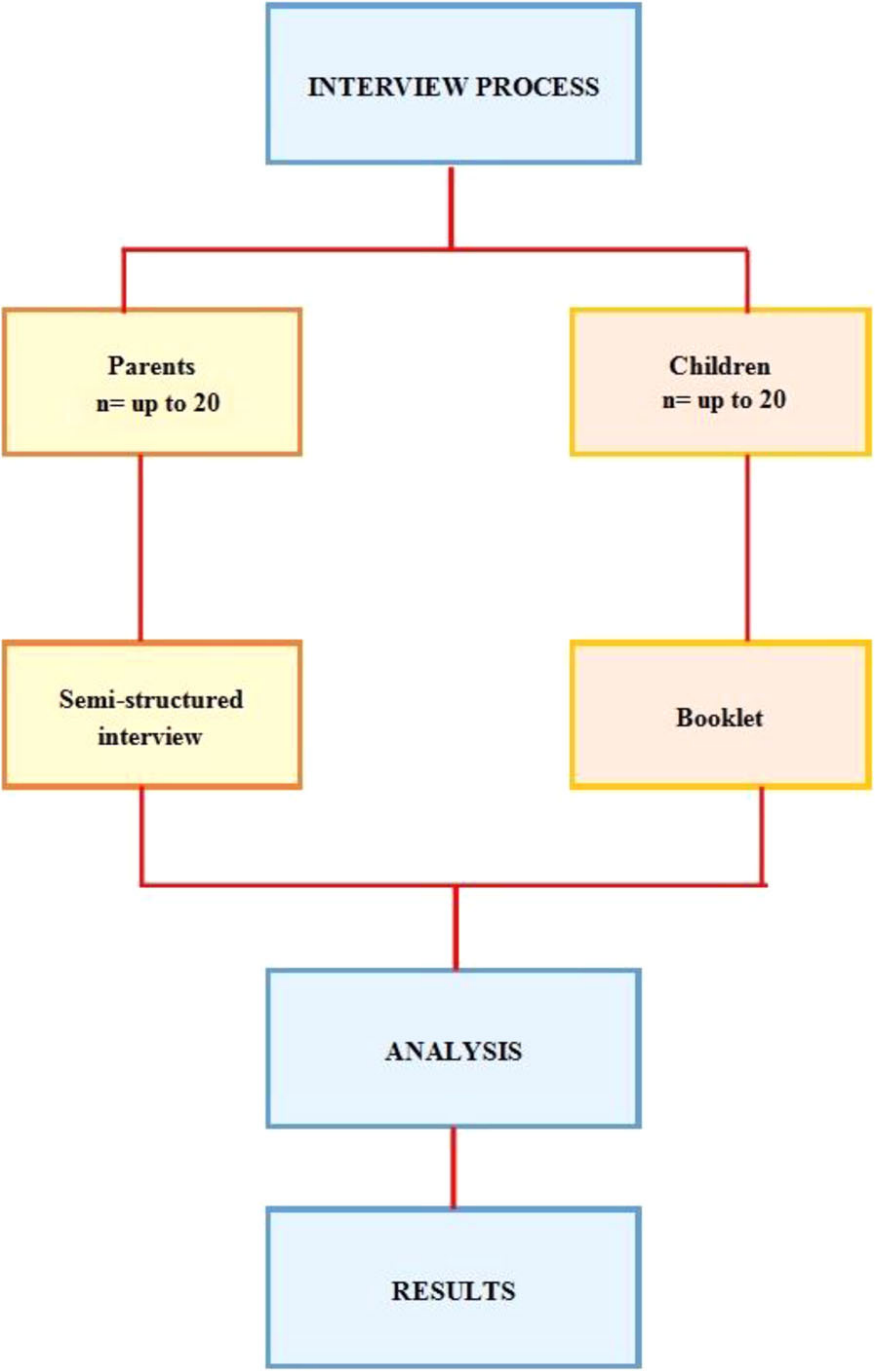
*Schematic* of the interview process

In order to determine the PROs for children with Perthes’ disease, the process will include two stages (Fig. 1):

1. Parents will be interviewed through a semi-structured interview process;
2. Children, with the help of the parents and/or of the interviewers (if needed), will complete a bespoke booklet to report their PROs. This booklet was initially designed with the help of two families affected by Perthes’ disease to ensure that it was sufficient to extract all of the relevant information. The booklet is used as a prompt to develop further discussion with the children.

Sample size will ensure insight into a diverse range of parent and child perspectives. We aim to recruit up to 40 participants, 20 with parents and 20 with their child with Perthes’ disease. The sample size estimation is based on general qualitative research guidelines [18] and it will be deemed complete when there will be agreement that saturation is reached, with no new outcome domains generated. The sample will purposively select a range of children aged 5–16 years, both boys and girls, at different stage of the disease (pre or post surgery, or treated with conservative approaches). The aim is to provide a richness in perspectives while remaining feasible within resource constraints. Data representing a variety of perspectives and from a diverse sample help to enhance the credibility of findings by demonstrating that the researcher has sought to present a balanced picture and not favoured one particular viewpoint or perspective [19]. Participants will be selected from patients attending Alder Hey Children’s Hospital Liverpool (UK), from members of the Perthes’ Association (UK) and via families known to the International Perthes’ Disease Study Group (IPSG).

Inclusion into this part of the study for children (and their parents) are related to the history of Perthes’ disease in the child (irrespective of the current stage of disease and treatment method) and the ability to be conversant in English.

#### Interview format

Among parents, a semi-structured interview will be used. Informed consent will be collected from the participants before the interview. The parent(s) of each child will be interviewed in a session that will last approximately 30 min. The interview will comprise a series of open-ended questions on their experiences and impact of the Perthes disease on their everyday life. The interviews aim to collect participants’ experience of the disease and the impact of Perthes’ disease on their lives, evaluating the daily needs that they have to deal with. The questions will investigate areas such as impact of the disease on patients and related family, the importance of clinical management, the impact of the disease on daily living activities and sport/recreational activities. Thus, based on our pilot work, the interview will be directed to the importance of defining key outcomes in the treatments and identifying possible outcomes in the management of Perthes’ disease. In the children’s group, a booklet (Additional file 2: Appendix S2) including questions related to Perthes’ disease and its influence in the child daily life, will be completed by each child, with the help of the interviewers where needed. The booklet aims to be a prompt for further discussion involving children and contains questions related to pain, hip mobility, related influence of the disease in the daily activities and effects of the treatment(s), explained through the use of emoji to ensure ease of completion. The final part of the booklet includes a personal description of a recent bad day and good day experienced by the child. This last part will be transcribed in children aged < 8 years (which will be helped by the interviewers) and recorded as an open-question interview in children aged > 8 years. The booklet completion process takes no longer than 30 min.

Consultation with the Health Research Authority deemed this study a service evaluation project with no requirement for ethical approval (reference 60/89/81). Informed consent will be assumed if participants agree to fill in the survey. A consent form indicating informed consent will be signed by parents to agree participation in the interview and allow voice recording of the interviews.

#### Interview analysis

All interviews will be recorded and transcribed; then, the transcripts and the recordings will be analysed in line with the qualitative approach following the National Centre for Research Methods guidelines [20].

The process of analysis of the qualitative data will summarise and define the key outcomes based on the stakeholders’ opinion.

### Identification of key outcomes to clinicians

#### Overview

**Fig. 2:**
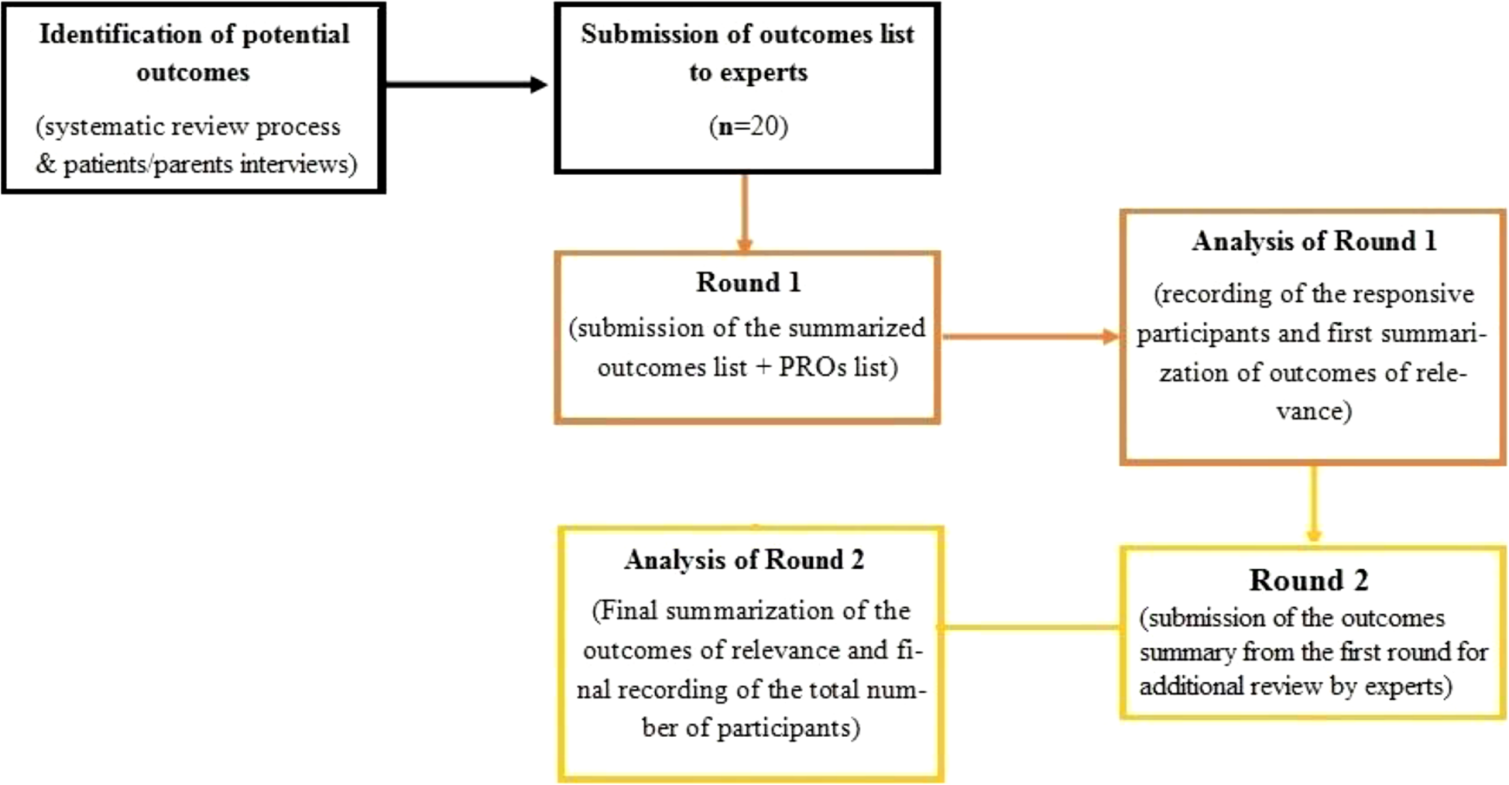
*Schematic* summary of Delphi Survey Process

A Delphi survey [21] (Fig. 2) will be conducted to identify the key outcomes important to orthopaedic surgeons, GPs and physiotherapists. The Delphi approach is a consensus technique that involves a series of questionnaires administered to target experts in the investigated area, which answer in an anonymous way in order to reduce reciprocal influences and bias [21].

#### Identification of potential outcomes

A complete list of all the outcomes present in the literature will be made following the approach of the systematic review described in this protocol. Additional outcomes will be included following the PROs obtained by the patients/parents’ interviews. Each outcome will be listed both individually and by domain.

#### Participants

Previous studies have indicated that a sample size of at least 20 clinicians is adequate in order to achieve the main goals of COS studies [22]. Participants will be those with experience of managing children with Perthes’ disease. This group of experts will include orthopaedic surgeons, GPs and physiotherapists, including both UK experts and an overseas experts group. The clinicians involved in the study will be selected through the British Society of Children’s Orthopaedic Surgery (BSCOS) and the International Perthes’ Disease Study Group (IPSG). Participants will be contacted and invited to participate in the survey by email using a bespoke COS Delphi management tool.

#### Delphi survey

The survey will be based on two stages (rounds). Clinicians involved in the study will have a three-week time period to complete each stage of the survey.

#### Delphi round 1

The electronic data collection form will seek details of participants’ demographic data (participant name, clinical role, place of work and contacts), seek the important list of selected outcomes (from the review and the patients/parents’ interviews) (to be graded on a score of 1–9 with ‘1–3 = not relevant’; ‘4–6 = important but not critical’; and ‘6–9 = extremely relevant’) and will give the possibility to add additional outcomes considered of importance (and related scores) not listed in the list.

#### Analysis of Delphi round 1

The analysis of the data will summarise the outcomes considered most important. Additional outcomes added by the clinicians will be reviewed by two assessors (DGL and WJL) in order to ensure that they do not refer to outcomes already listed. The number of the invited participants that respond to the survey will also be recorded.

#### Delphi round 2

At the second stage, participants involved in round 1 of the Delphi survey will be able to see the summary of the data obtained in round 1, asking them to review again the list of outcomes, considering if the outcomes present in the summarised list have to be classified as relevant or not.

Participants that do not respond to round 1 will be excluded in round 2.

#### Analysis of Delphi round 2

The total number of participants invited to participate and do participate in round 2 will be recorded. The distribution of scores will be summarised. In the summary of the percentage agreement, each individual outcome will be classified as ‘consensus in’, ‘consensus out’ or ‘no consensus’ based on the percentage.

### Consensus meeting

The final stage of the study will be based on a consensus meeting between a selected group of clinicians and a selected group of patients/parents (for a total of 24 participants, adhering to the OMERACT guidelines for the consensus meeting structure [14]).

Before the meeting, the patients/parents group will be able to review the outcomes selected by the clinicians during the Delphi survey and these data will be discussed during the consensus meeting.

#### Definition of consensus

Following the GRADE guidelines [23], in order to define consensus, outcomes inclusion will be indicated as the agreement by the vast majority (> 70% of the group) of the ‘extremely relevant’ (7–9 points range) of the discussed outcomes, with only a minority (< 15% of the group) of participants that consider it as ‘not relevant’ (1–3 points range). Consensus for outcomes exclusion will be indicated as the agreement by the vast majority (> 70% of the group) of the ‘not relevant’ (1–3 points range) of the discussed outcomes, with only a minority (< 15% of the group) of participants that consider it as ‘extremely relevant’ (7–9 points range).

## Discussion

The evaluation of literature on Perthes’ disease shows a clear lack of common outcome measures reported among different studies in the literature. This lack of a COS impacts the ability to produce meaningful research and inhibits the ability to compare research findings in order to clearly define the management guidelines for Perthes’ disease. Thus, a clear definition and implementation of a COS is required in order to help future researchers identify the primary outcome measures in their studies in order to increase the quality and the clinical application of the results obtained.

## Trial status

The systematic review and the patients’ recruitment for the interview process is currently ongoing.

## Search strategies

PubMed search strategy: 1 January 1990 to 1 January 2017

1. ‘Femur Head Necrosis’ [MeSH]
2. Osteonecrosis [MeSH]
3. (Perthe* OR Legg-Calv*-Perthe* OR Legg-Perthe* OR Calv*-Perthe*)
4. (Perthe* AND Legg-Calv*-Perthe* AND Legg-Perthe* AND Calv*-Perthe*)
5. (#1 OR #2 OR #3 OR #4) AND Hip*
6. (#5) AND (Child* OR Infant*)

Cochrane CENTRAL search strategy: 1 January 1990 to 1 January 2017

1. MeSH descriptor: [Femur Head Necrosis] explode all trees
2. MeSH descriptor: [Osteonecrosis] explode all trees
3. (TITLE-ABSTRACT-KEYWORDS) Perthe* OR Legg-Calv*-Perthe* OR Legg-Perthe* OR Calv*-Perthe*
4. (TITLE-ABSTRACT-KEYWORDS) Perthe* AND Legg-Calv*-Perthe* AND Legg-Perthe* AND Calv*-Perthe*
5. (TITLE-ABSTRACT-KEYWORDS) (#3 OR #4) AND Hip*
6. (TITLE-ABSTRACT-KEYWORDS) (#5) AND (Child* OR Infant*)

Web of Science search strategy: 1 January 1990 to 1 January 2017

1. (TOPIC) ‘Femur Head Necrosis’
2. (TOPIC) Osteonecrosis
3. (TOPIC) Perthe* OR Legg-Calv*-Perthe* OR Legg-Perthe* OR Calv*-Perthe
4. (TOPIC) Perthe* AND Legg-Calv*-Perthe* AND Legg-Perthe* AND Calv*-Perthe*
5. (TOPIC) (#1 OR #2 OR #3 OR #4) AND Hip*
6. (TOPIC) (#5) AND (Child* OR Infant*)

## Additional files

Additional file 1: Appendix S1. Systematic review data extraction form. (DOCX 20 kb)

Additional file 2: Appendix S2. Children’s booklet. (DOCX 242 kb)

## Abbreviations

BSCOS: British Society of Children’s Orthopaedic Surgery
COMET: Core Outcome Measures in Effectiveness Trials
COS: Core outcomes set
GRADE: Grading of Recommendations Assessment, Development and Evaluation
IPSG: International Perthes’ Disease Study Group
OMERCAT: Outcome Measures in Rheumatology
PICO: Population Intervention Comparison Outcomes
PRISMA: Preferred Reporting Items for Systematic Reviews and Meta-Analyses
PRO: Patient-reported outcome
ROM: Range of motion
RTC: Randomised controlled trial

## References

[1] Perry DC, Skellorn PJ, Bruce CE (2016). The lognormal age of onset distribution in Perthes’ disease: an analysis from a large well-defined cohort. J Bone Joint Surg Br.

[2] Perry DC, Bruce CE, Pope D, Dangerfield P, Platt MJ, Hall AJ (2012). Comorbidities in Perthes’ disease: a case control study using the general practice research database. J Bone Joint Surg Br.

[3] Perthes’ WO (2009). Disease in Norway a prospective study on 425 patients. Acta Orthop Suppl.

[4] Perry D, Bruce C (2011). Hip disorders in childhood. Surgery.

[5] Perry D, Hall A (2011). The epidemiology and etiology of Perthes’ disease. Orthop Clin.

[6] Stulberg SD, Cooperman DR, Wallensten R (1981). The natural history of Legg-calve-Perthes’ disease. J Bone Joint Surg Am.

[7] Perthes Association UK. 2016. https://www.perthes.org.uk. Accessed May 2017.

[8] Karimi MT, McGarry T. A comparison of the effectiveness of surgical and nonsurgical treatment of Legg-calve-Perthes’ disease: a review of the literature. Adv Ortho. 2012;2012:1–7. 10.1155/2012/490806 PMC3431042 22953066

[9] The Core Outcome Measures in Effectiveness Trials (COMET) Initiative. 2016. http://www.comet-initiative.org. Accessed May 2017.

[10] Haywood KL, Griffin XL, Achten J, Costa ML (2014). Developing a core outcome set for hip fracture trials. J Bone Joint Surg.

[11] Singh JA, Dohm M, Choong PF (2017). Consensus on draft OMERACT Core domains for clinical trials of total joint replacement outcome by orthopaedic surgeons: a report from the international consensus on outcome measures in TJR trials (I-COMiTT) group. BMC Musculoskelet Disord.

[12] Ouzzani M, Hammady M, Fedorowicz Z, Elmagarmid A (2016). Rayyan — a web and mobile app for systematic reviews. Syst Rev.

[13] Moher D, Liberati A, Tetzlaff J, Altman DG, The PRISMA Group (2009). Preferred reporting items for systematic reviews and meta-analyses: the PRISMA statement. PLoS Med.

[14] Boers M, Kirwan JR, Wells G, Beaton D, Gossec L, D’Agostino MA (2014). Developing Core Outcome Measurement Sets for Clinical Trials: OMERACT Filter 2.0. J Clin Epidemiol.

[15] Dorman SL, Shelton JA, Stevenson RA, Linkman K, Kirkham J and Perry DC.Management of medial humeral epicondyle fractures in children: a structured review protocol for a systematic review of the literature and identification of a core outcome set using a Delphi survey. Trials. 2018;19:119. 10.1186/s13063-018-2472-3 PMC5819271 29458402

[16] Long AF, Dixon P (1996). Monitoring outcomes in routine practice: defining appropriate measurement criteria. J Eval Clin Pract.

[17] Macefield RC, Jacobs M, Korfage I, Nicklin J, Whistance RN, Brookes ST (2014). Developing core outcomes sets: methods for identifying and including patient-reported outcomes (PROs). Trials.

[18] Baker SE, Edwards R. How many qualitative interviews is enough? NCRM; 2012. Discussion paper. http://eprints.ncrm.ac.uk/2273/4/how_many_interviews.pdf. [Accessed May 2018].

[19] Rubin HJ, Rubin I (2005). Qualitative interviewing: the art of hearing data.

[20] Malterud K, Siersma VD, Guassora AD. Sample size in qualitative interview studies: guided by information power. Qual Health Res. 2015:1–8. 10.1177/1049732315617444 26613970

[21] Smith J, Firth J (2011). Qualitative data analysis: application of the framework approach. Nurse Res.

[22] Hsu C, Sandford B (2007). The Delphi technique: making sense of consensus. Pract Assess Res Eval.

[23] Schünemann H, Brożek J, Guyatt G, Oxman A, editors. GRADE handbook for grading quality of evidence and strength of recommendations. Updated October 2013. The GRADE Working Group, 2013. Available from http://gdt.guidelinedevelopment.org/app/handbook/handbook.html. [Acessed May 2018].

