# Supplementary material for: The outcomes of Perthes’ disease of the hip: a study protocol for the development of a core outcome set
Source: Trials. 2018 Jul 13;19:374. doi: 10.1186/s13063-018-2695-3 (PMC6044030; doi:10.1186/s13063-018-2695-3)
Supplement: Supplementary file 1 — Appendix S1. Systematic review data extraction form. (DOCX 20 kb) [file 13063_2018_2695_MOESM1_ESM.docx]

**Appendix S1 – Systematic Review Data Extraction Form**

| **Reference:** |  |
| --- | --- |
| **Objective of the study:** |  |
| **Were cases identified prospectively?** |  |
| **Location:** |  |
| **Study type:** |  |
| **Population:** |  |
| Mean age at time of the intervention (+SD/ SE if available) |  |
| **No. of patients within the study?** |  |
| **No. of hips within study?** |  |
| **CONSERVATIVE** – give details of the regimen (i.e. bracing) |  |
| **SURGICAL** –give the details of the surgical intervention (i.e. shelf osteotomy) |  |
| **Outcomes measured**  **Radiographic Evaluation**  **Pain**  **Range of Motion (ROM)**  **Gait impairments**  **Other (specify)**  **Outcome tools:**  **Functional assessment:**  **Primary outcome measurement(s):** | |
| **Follow up:** |  |
| **Notes:** |  |
| **Author’s Contact** |  |
