# Supplementary material for: The outcomes of Perthes’ disease of the hip: a study protocol for the development of a core outcome set
Source: Trials. 2018 Jul 13;19:374. doi: 10.1186/s13063-018-2695-3 (PMC6044030; doi:10.1186/s13063-018-2695-3)
Supplement: Supplementary file 2 — Appendix S2. Children’s booklet. (DOCX 242 kb) [file 13063_2018_2695_MOESM2_ESM.docx]

**Appendix S2 – Children Booklet**


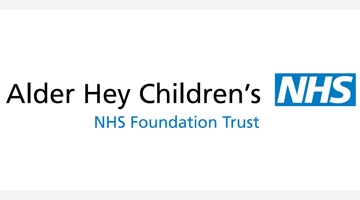

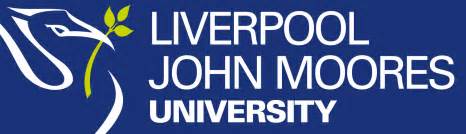

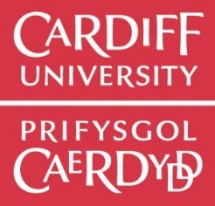

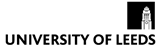


**HOW PERTHES AFFECTS**

**WHAT YOU DO AND HOW YOU FEEL**

Dear Parent,

We are very interested in how your son whose has Perthes affects what he does and how he feels. We have drawn up the following short booklet to help him to tell us, in his own words, how his bad hip affects what he does and how he feels.

Many thanks for all your help.

Dr Tina Gambling (Cardiff University) and Prof Andrew Long (University of Leeds)

**Introduction**

This is new booklet to help us find out how your Perthes affects what you do and the way you feel.   We have tried to make these questions as easy as possible to answer.  Please try to answer all the questions. If you need your mum or dad to help you, that is fine.

In the tables on the next two pages, we are asking you what you can and cannot do and how you feel on a typical *good* day and a typical *bad* day. Please choose the smiley face that best matches how you feel or what you are able to do.

| Answer | Meaning |
| --- | --- |
| 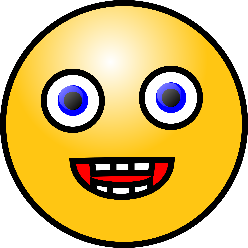 | I am very happy; I can do what I want without any pain. |
| 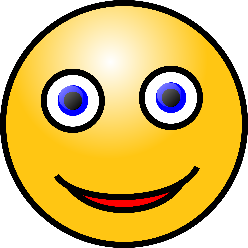 | I am quite happy; I can do most of what I want to but with some pain. |
| 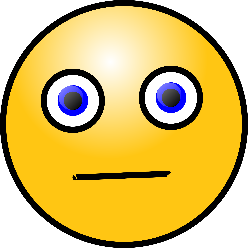 | It is okay. I could do some of what I wanted with some pain which sometimes got worse. |
| 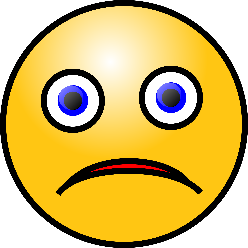 | I am quite sad. I cannot most of what I want. I am in too much pain. |
| 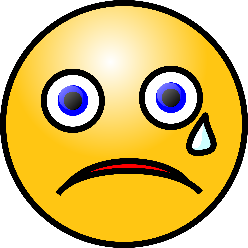 | I am very sad. The pain is very bad. I cannot do things and I normally do when I am not in so much pain. |

**Example**:

On a *good* day, I was able to do everything I wanted to, play outside and with my friends.

Your answer might look like this.


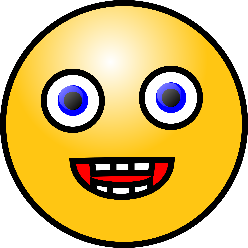


On a *bad* day, I was unable to do what I wanted. The pain was too much. I could not go to school and just hoped some of my friends or my brother or sister might come and sit with me.

Your answer might look like this.


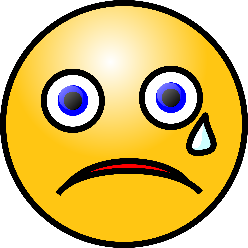


**Now please turn to the next page of the booklet and try to tell us about the ways that your Perthes affects what you do and how you feel.**

**Can you tell us about …**

| **Question** | **On a *typical good* day…** | **On a *typical bad* day…** |
| --- | --- | --- |
| How painful is your hip? |  |  |
| I still have to take some painkillers  I have to take lots of painkillers |  |  |
| I am able to do lots of things |  |  |
| I can see and play with my friends |  |  |
| I am able to go to pre-school or school |  |  |
| I am able to sleep well at night |  |  |
| My hip does not make me feel so sad  My hip makes me feel so sad |  |  |
| My hip does not make me feel fed up  My hip makes me feel so fed up |  |  |

**Now, can you write us a short story about how your hip affects you…?**

It would be really helpful if you would write down how your hip affects you. Can you do this for a *recent good day* and then again for a recent bad day?

**ON A *RECENT GOOD* DAY…**

Can you tell us about a recent *good* day, when you have been able to do lots of thing you would normally do and had little pain? Tell us, if you can what you did from when you woke up and got up to when you went to bed. Maybe write it as a short story and draw us a picture too. Do ask your mum or dad to help if you want.

Please write what you want here and on another sheet of paper if you like.

**When I woke up and got up in the morning….**

**When I went to school …**

**When I came back from school until I went to bed …**

**And now can you write us a short story about how your hip affects you…**

**ON A *RECENT BAD* DAY…**

Can you tell us about a recent *bad* day, when your hip was very painful and stopped you doing what you would like to or would normally on a good day be able to do. Tell us, if you can, what you did from when you woke up and got up to when you went to bed. Maybe write it as a short story and draw us a picture too. Do ask your mum or dad to help if you want.

Please write what you want here and on another sheet of paper if you like.

**When I woke up and got up in the morning….**

**When I went to school …**

**When I came back from school until I went to bed …**

**YOU ARE NOW FINISHED. THANK YOU.**

**PLEASE GIVE THIS TO BACK TO US**
